# Novel Phenoxazinones as potent agonist of PPAR-α: design, synthesis, molecular docking and in vivo studies

**DOI:** 10.1186/s12944-018-0764-y

**Published:** 2018-05-22

**Authors:** David I. Ugwu, Uchechukwu C. Okoro, Narendra K. Mishra, Sunday N. Okafor

**Affiliations:** 1 Medicinal Chemistry unit, Department of Pure and Industrial Chemistry, University of Nigeria, Nsukka, 410002 Nigeria; 2 Department of Chemistry, Indian Institute of Technology, Kanpur, India; 3 Department of Pharmaceutical and Medicinal Chemistry, University of Nigeria, Nsukka, Nigeria

**Keywords:** Carboxamide, Cardiac disease, Cholesterol, Molecular docking, Sulphonamides, Synthesis

## Abstract

**Background:**

The use of statin, a 3-hydroxy-3-methylglutaryl coenzyme A reductase inhibitor for the treatment of dyslipidemia has been associated with dose limiting hepatoxicity, mytotoxicity and tolerability due to myalgias thereby necessitating the synthesis of new drug candidates for the treatment of lipid disorder.

**Methods:**

The reaction of appropriate benzenesulphonamide with substituted phenoxazinone in the presence of phenylboronic acid gave the targeted compounds. The molecular docking study were carried out using autodock tool against peroxisome proliferator activated receptor alpha. The in vivo lipid profile were assayed using conventional methods. The kidney and liver function test were carried out to assess the effect of the derivatives on the organs. The LD_50_ of the most active derivatives were determined using mice.

**Results:**

The targeted compounds were successfully synthesized in excellent yields and characterized using spectroscopic techniques. The results of the molecular docking experiment showed that they were good stimulant of peroxisome proliferator activated receptor alpha. Compound **9f** showed activity at *Ki* of 2.8 nM and binding energy of 12.6 kcal/mol. All the compounds tested reduced triglyceride, total cholesterol, low density lipoprotein cholesterol and very low density lipoprotein cholesterol level in the mice model. Some of the reported compounds also increased high density lipoprotein cholesterol level in the mice. The compounds did not have appreciable effect on the kidney and liver of the mice used. The LD_50_ showed that the novel compounds have improved toxicity profile.

**Conclusion:**

The synthesis of fifteen new derivatives of carboxamides bearing phenoxazinone and sulphonamide were successful. The compounds possessed comparable activity to gemfibrozil. The reported compounds had better toxicity profile than gemfibrozil and could serve as a replacement for the statins and fibrate class of lipid agents.

## Background

**Fig. 1:**
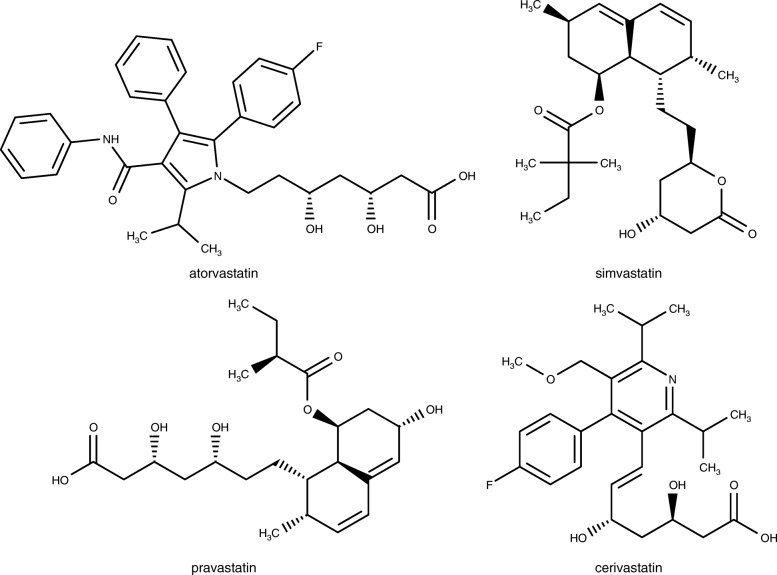
Statins

High level of low density lipoprotein cholesterol (LDLC), triglycerides and low levels of high density lipoprotein cholesterol (HDLC) are considered to be among the predominant risk factor in coronary heart disease [1]. Hypercholesterolemia is a common disorder which is known as main cause of coronary heart disease (CHD) [2]. This disease is recognized as cause of the most of the deaths in developed countries. High levels of total cholesterol (TC), triglycerides (TG), and low density lipoprotein-cholesterol (LDLC) have been implicated as contributing risk factors in progress of CHD and atherosclerosis [3]. Accordingly, different lipid lowering drugs have been applied for the treatment of hypercholesterolemia [4]. Statins are the effective hypocholesterolemic drugs which competitively inhibit the activity of 3-hydroxy-3-methylglutaryl-coenzyme A (HMG-CoA) reductase, the rate limiting enzyme in cholesterol biosynthesis [5]. Heart disease is a major cause of mortality and morbidity in the world. The main risk factors, such as family history and age cannot be changed. However, other risk factors including, obesity, diabetes, smoking, diet, high blood pressure, total cholesterol (TC), low-density lipoprotein cholesterol (LDLC), and low levels of high-density lipoprotein cholesterol (HDLC) can be changed or treated [6, 7]. These risk factors can be controlled and corrected by diet, exercise, hypolipidemic drugs and herbal medicine [8]. Currently, the most common method to treat dyslipidemia is the use of statins typified by simvastatin and atorvastatin (Fig. 1) which are HMG-CoA reductase inhibitors. The widespread clinical use of the statins is accompanied by potential dose-limiting hepatoxicity and mytotoxicity [9]. Cerivastatin, one of the second generation statins was withdrawn from the world market in 2001 due to its adverse effects [10]. The work of Ference et al. on Mendelian randomized trials further highlights the need for new drugs for the treatment of coronary heart disease linked to cholesterol [11].

**Fig. 2:**
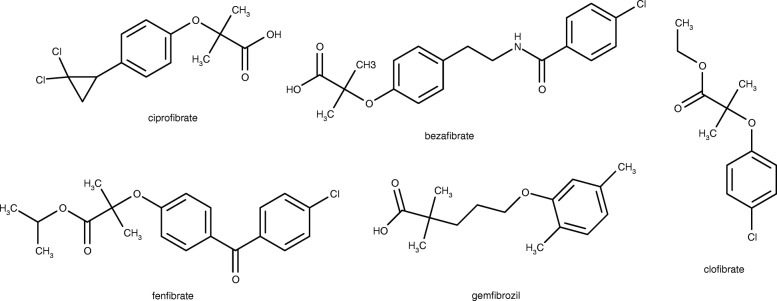
Fibrates

The fibrate classes of lipid lowering drugs typified by gemfibrozil (Fig. 2) are selective activators of α-isotype of the receptors peroxisome proliferator activated receptor (PPAR) [12, 13]. PPAR-α agonists lower triglyceride levels and increase HDLC level in hyperlipidemic patients [14] and reduce the risk of coronary heart disease in patients with low HDLC levels [15]. Clofibrate was the first fibrate to be developed. Researches targeted on development of lipid lowering agent were revolutionized following the discovery of the other fibrates such as ciprofibrate, bezafibrate, fenofibrate and gemfibrozil (Fig. 2). However, the reported proliferation of peroxisome leading to hepatomegaly and formation of tumour in the liver of rodents associated with fibrates is worrisome [16–20].

To address the problems of toxicities associated with statins and fibrates, ezetimibe was launched in 2002 as a new drug which inhibits cholesterol absorption in the intestine without affecting the absorption of triglycerides [21]. Ezetimibe inhibits free cholesterol uptake from the intestine and as such reduces plasma LDLC levels. However, the activity of ezetimibe was not comparable to the statins and fibrates, thereby necessitating the development of new class of lipid lowering agent that will retain the high activity of fibrates and statins and an improved toxicity profile.

Although there is no report to our knowledge on sulphonamides used as lipid lowering agents, the reported wide biological activities of sulphonamide aroused the curiosity in synthesizing novel sulphonamides and testing them for lipid lowering activity. Again, most of the statins and fibrate class of lipid lowering agents contains a carboxamide and/or carboxylic acid group which underscores the importance of carboxamide functionality in lipid lowering agents.

We therefore report herein the first class of phenoxazinone derivatives bearing sulphonamide and carboxamide functionalities as potential lipid lowering agents. This research exploited the relative stability of sulphonamides, their wide pharmacological applications and ease of synthesis in combination with the reported lipid lowering activity of carboxamides. The need for new lipid lowering agents with comparable activity with the statins and fibrates, ease of synthesis and low toxicity profile motivated this research.

## Methods

### Instrumentation

All reactions requiring inert atmosphere were carried out under nitrogen atmosphere. Drying of solvents was achieved using molecular sieve for 48 h. All reagents were purchased from commercial suppliers, Aldrich, Merck, Fluka, Avra, SD fine and Alfa Aesar. Thin layer chromatography was carried out using silica plates purchased from Avra. The plates were visualized under UV light (popular India). FT-IR spectroscopy of the compounds was run in PerkinElmer Spectrum version 10.03.06 and the bands presented in wavenumber. Proton and carbon-13 NMR spectroscopy were run in DMSOd_6_ and CD_3_OD, unless otherwise stated on either Jeol 500 MHz or 400 MHz. The chemical shifts were reported in part per million with reference to tetramethylsilane. High Resolution Mass spectrometry (HRMS) was carried out using electrospray ionization-time of flight (ESI-TOF) mass spectrometer (Aerodyne Research Inc. USA), sodium formate was used as the calibrant. All experiments were carried out at Prof. Sandeep Verma’s Laboratory, department of Chemistry, Indian Institute of Technology, Kanpur. Melting points were determined using digital melting point apparatus (Stuart, SMP20) and were uncorrected. The biological studies were carried out at the Department of Biochemistry, University of Nigeria, Nsukka under the guidance of Mr. OGB.

### Synthesis of substituted benzenesulphonamides (3a-p)

Sodium carbonate (Na_2_CO_3_, 1.590 g, 15 mmol) was added to a solution of amino acids (**2a-h,** 12.5 mmol) in water (15 mL) with continuous stirring until all the solutes had dissolved. The solution was cooled to − 5 °C and the appropriate benzenesulphonyl chloride (**1a-c,** 15 mmol) was added in four portions over a period of 1 h. The slurry was further stirred at room temperature for about 4 h. The progress of the reaction was monitored using TLC (MeOH/DCM, 1:9). Upon completion, the mixture was acidified using 20% aqueous hydrochloric acid to pH 2. The crystals was filtered via suction and washed with pH 2.2 buffer. The pure products (**3a-x**) were dried over self-indicating fused silica gel in a desiccator [22].

### Synthesis of *N*-benzoyl derivatives of Benzenesulphonamides (5a-f and i-m)

Appropriate benzenesulphonamides (**3a-f** and i**-m**, 1.0 mmol) was dissolved in NaOH (10%, 10 mL) in a 50 mL round bottom flask. Benzoyl chloride (**4,** 1.1 mmol, 0.2 mL) was transferred into the solution of appropriate benzenesulphonamides and stirred at room temperature. The reaction progress was monitored by TLC (3% MeOH/CH_2_Cl_2_) to the disappearance of the benzenesulphonamides spot. Upon completion, the solution was transferred into a beaker containing crushed ice and then acidified to pH of 3 with concentrated hydrochloric acid. The solid was collected via suction filtration and transferred into a beaker containing CCl_4_ (10 mL) and covered with watch glass boiled for 10 min. The mixture was allowed to cool slightly and then filtered. The products (**5a-f** and i-**m**) obtained were washed with 10–20 mL of CCl_4_ and dried over fused self-indicating silica gel in a dessicator [22].

### Synthesis of 4-amino-11-chloro-2-sulfanyl-10*H*-12-oxa-1,3,5-triazatetraphen-10-one (8)

5,6-Diamino-2-sulphanylpyrimidin-4-ol (**6,** 2.47 g), 2,3-dichloro-1,4-dihydronaphthalen-1,4-dione (**7,** 2.27 g), sodium acetate (0.36 g) and benzene (60 mL) mixed with dimethyl formamide (30 mL) were charged into 250 mL two-necked round bottomed flask fitted with short magnetic stirring bar and a reflux condenser. The mixture was stirred while heating on a water bath at 70–75 °C for 8 h. The colour of the reaction mixture changed from light brownish green to yellowish red and intense red as the reaction progressed. At the end of 8 h, the reaction mixture was filtered and cooled in ice overnight and filtered to obtain a solid. Analytical sample was obtained by column chromatography using benzene as the eluting solvent followed by recrystallization to obtain the target compound.

### Phenylboronic acid catalysed Amidation reaction of carboxylic acid and 4-amino-11-chloro-2-sulfanyl-10*H*-12-oxa-1,3,5-triazatetraphen-10-one derivatives (9a-o)

*N*-benzoylated-substituted-benzenesulphonamides (1 mmol), was dissolved in dry toluene (50 mL). Phenylboronic acid (0.1 mmol) was added to the above solution and then 4-amino-11-chloro-2-sulphanyl-10*H*-12-oxa-1,3,5-triazatetraphen-10-one (1 mmol) was added. The mixture was dissolved using ultrasonication and then refluxed for an average of 10 h using Dean-Stark apparatus for azeotropic removal of water. Upon completion of reaction (TLC monitored), the mixture was allowed to cool to ambient temperature and the amides precipitated via the addition of 30 mL of n-hexane. The crystals were washed with n-hexane after filtration using suction. The products were dried over fused silica gel.

### Molecular docking experiment

#### Preparation of ligands

**Fig. 3:**
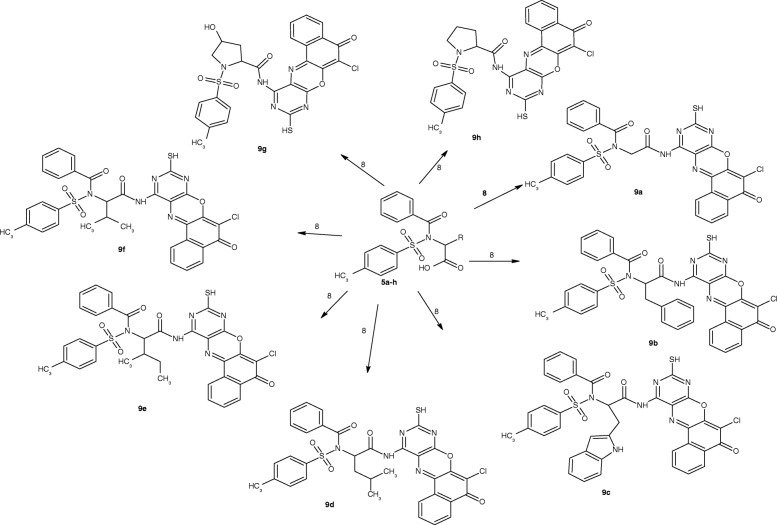
4-methylbenzenesulphonamides derivatives (9**a**-**h**)

**Fig. 4:**
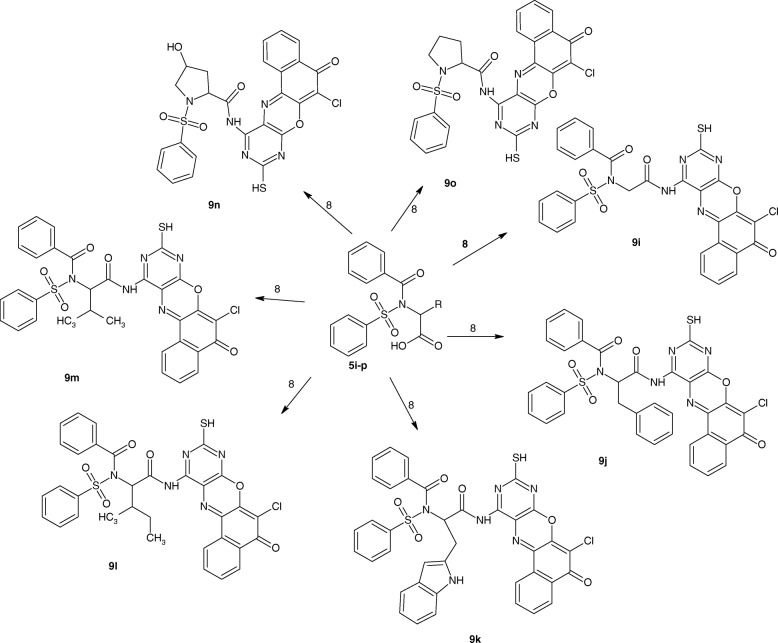
benzenesulphonamides derivatives (9**i**-**o**)

ACD/ChemSketch 2015 (Ref: ACD/Structure Elucidator, version 15.01, Advanced Chemistry Development, Inc., Toronto, ON, Canada, www.acdlabs.com, 2015.) was used to draw the structures of compounds 9a-o (Figs. 3 and 4) and also convert them to 3D formats.

#### Preparation of protein targets

The 3D structure of the peroxisome proliferator-activated receptor alpha (PPAR-α, pdb code 2p54) was retrieved from the RCSB Protein Data Bank (PDB) (www.rcsb.org/pdb/home/home.do). All bound ligands, cofactors, and water molecules were removed from the proteins using Discovery Studio Visualizer v16. 1.0. 15,350.

All file conversions required for the docking study were performed using the open source chemical toolbox Open Babel version 2.3.2 (www.openbabel.org).

#### Molecular docking experiments

In our effort to identify potential lipid lowering lead(s) among compounds **9a–o,** we carried out docking calculations using Autodock v4.0.1 into the 3D structure of the catalytic sites of 2p54,. The Gasteiger charge calculation method was used and partial charges were added to the ligand atoms prior to docking. The Lamarckian genetic algorithm (LGA), which is available in Auto Dock, was employed [23]. Finally, Auto Dock was used to calculate the binding free energy of a given inhibitor conformation in the macromolecular structure.

### In vivo lipid lowering activities determination

Male mice were kept on a 12 h light/dark cycle at a temperature of 22 ± 1 °C. After acclimatizing for one week in a cage, animals were randomly divided into three groups (*n* = 6):

**Group 1**: hypercholesterolemic group (received chow + 2% *w*/w cholesterol + 0.5% w/w cholic acid) [24].

**Group 2**: carboxamide (received hypercholesterolemic diet + 0.001% w/w carboxamide).

**Group 3**: control (chow only).

High density lipoprotein cholesterol (HDLC), triglyceride (TG) and total cholesterol (TC) levels were at the baseline prior to the experiment and were similar between animals. New carboxamides (**9a-o**) were dissolved in physiological saline and mixed with animal’s diet. An equivalent amount of saline was added to the diet of hypercholesterolemic and control groups. At the end of four weeks, mice were fasted for 12 h, and then anesthetized using CO_2_ and sacrificed. Blood was then collected from the heart and serum was separated by centrifugation at 3000 g for 10 min. All the dead mice were disposed in bio-safety containers in accordance with local standard protocols.

#### Determination of Total cholesterol concentration

Total cholesterol concentration was determined by the method of Allain et al. [25] using Randox kit.

#### Determination of high-density lipoproteins (HDL)-cholesterol concentration

The concentration of high-density lipoprotein (HDL) was determined by the method of Albers et al. [26] using Randox kit.

#### Determination of low-density lipoprotein (LDL)–cholesterol concentration

The concentration of Low density lipoprotein-Cholesterol was determined based on the Polyvinylsulphate method described by Demacker et al. [27].

#### Determination of triacylglycerol concentration

The concentration of triacylglycerol (TG) was determined by the method of Allain et al. [24] using Randox kit.

### Liver function tests (LFTs)

The liver function tests were carried out using aspartate aminotransferase (AST), alanine transaminase (ALT) and alkaline phosphatase (ALP) as biomarkers employing the methods of Reitman and Franke 1957 [28].

### Renal or kidney function test

Kidney function tests were carried out on the two most active derivatives (**9f** and **9 k**) using serum urea, creatinine and uric acid as biomarkers. The method reported by Kaplan and Teng 1982 [29] was used in the determination of urea and creatinine while that reported by Ochie and Kolhattar 2000, was used for the determination of uric acid [30].

### Determination of LD_50_ for the active anti-inflammatory compounds

Male mice were divided into various groups and test compounds were administered in various doses intraperitoneally. Following treatments, the animals were observed for up to 4 h continuously and were then kept under observation for 72 h. All behavioral changes and deaths during the observation periods were recorded. The percentage of death at each dose level was then calculated, converted to probits and the LD_50_ (μM/kg) values were calculated [31]. To observe the health status of the mice, they were monitored 4 times a day. Humane endpoints were used when the animal shows sign of weight loss, weakness accompanied by inability to get food, complete anorexia and convulsion for 24 h. For the purpose of ameliorating the suffering of the dying mice, CO_2_ euthanasia was applied. All the dead mice were disposed in bio-safety containers in accordance with local standard protocols. The mortality rate in each group was calculated according to the formula:

Mortality rate (%) = (the number of dead mice/the number of mice in the group) × 100.

## Results and discussion

### Synthesis of the target compounds

**Scheme 1:**
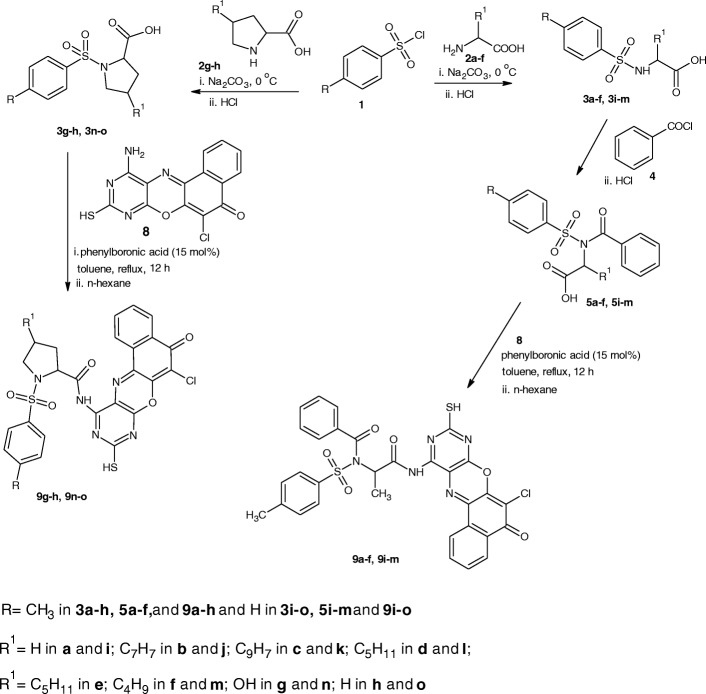
Synthesis of the target compounds (**9a-o**)

All the 4-amino-11-chloro-2-sulfanyl-10*H*-12-oxa-1,3,5-triazatetraphen-10-one derivatives (9a-o, Figs. 3 and 4) showed similar pattern of absorption in the FTIR, ^1^H NMR, ^13^C NMR and MS. Worthy of mention in the FTIR is the absorptions between 3405 and 3312 cm^− 1^ due to NH stretch, 2559–2468 cm^− 1^ due to SH stretch, 1763–1714 cm^− 1^ due to C=O of ketone, the bands between 1637 and 1619 cm^− 1^ due to C=N stretch, the band between 756 and 743 cm^− 1^ due to C-Cl stretch. These bands are indicative of successful coupling of 4-amino-11-chloro-2-sulfanyl-10*H*-12-oxa-1,3,5-triazatetraphen-10-one. In the proton NMR, the peak between 11.9–11.8 was indicative of the presence of SH proton. In the carbon-13 NMR spectra of the 4-amino-11-chloro-2-sulfanyl-10*H*-12-oxa-1,3,5-triazatetraphen-10-one Derivatives (9a-o)**,** the appearance of the ketonic carbonyl peak between 178.4–172.7, the azomethine carbon peak between 170.0–151.1 ppm seriously indicated the successful formation of the target molecules. The spectral analysis above in addition to the molecular ion peaks showed that there was successful coupling of the amino group of 4-amino-11-chloro-2-sulfanyl-10*H*-12-oxa-1,3,5-triazatetraphen-10-one with the carboxylic acid of the benzenesulphonamides. The summaries of the 4-amino-11-chloro-2-sulfanyl-10*H*-12-oxa-1,3,5-triazatetraphen-10-one derivatives (9a-o) are presented in Scheme 1.

### Molecular docking

We undertook molecular docking studies to gain more insight into the binding interactions between these compounds and the receptor (2p54).

**Fig. 5:**
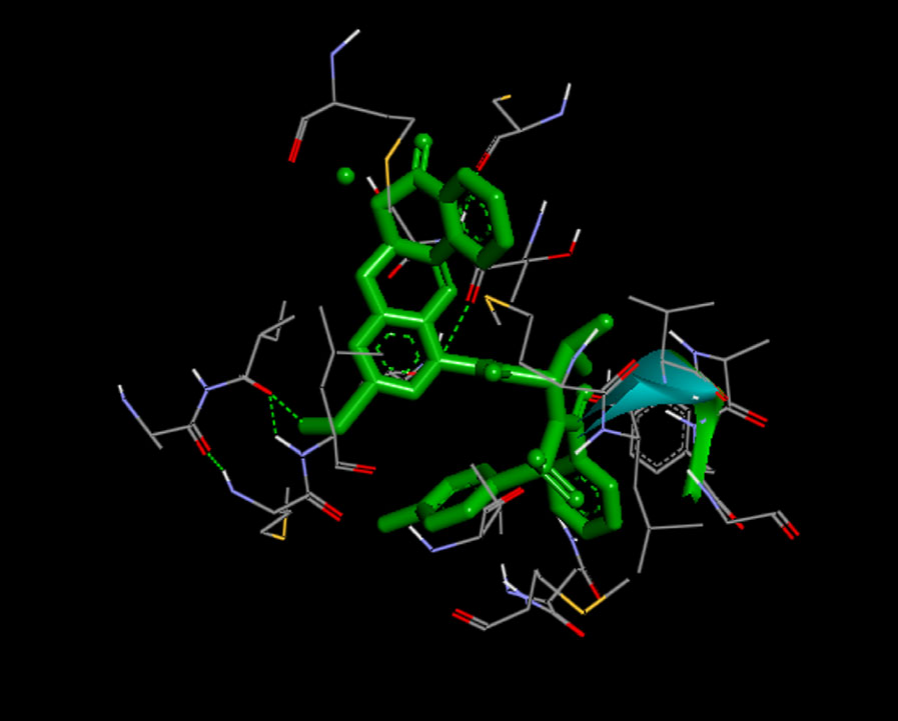
binding mode of 2p54_9f

**Fig. 6:**
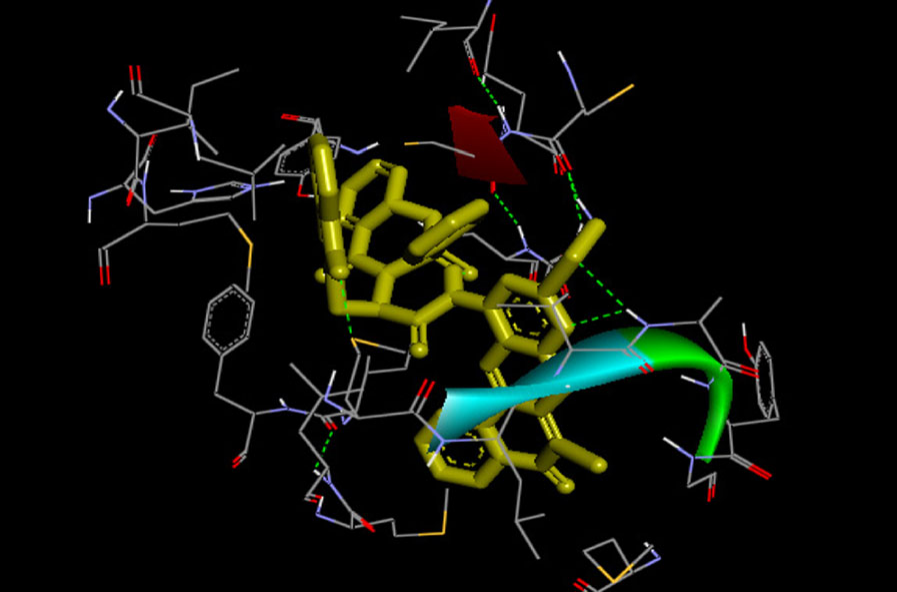
binding mode of 2p54_9 k

**Fig. 7:**
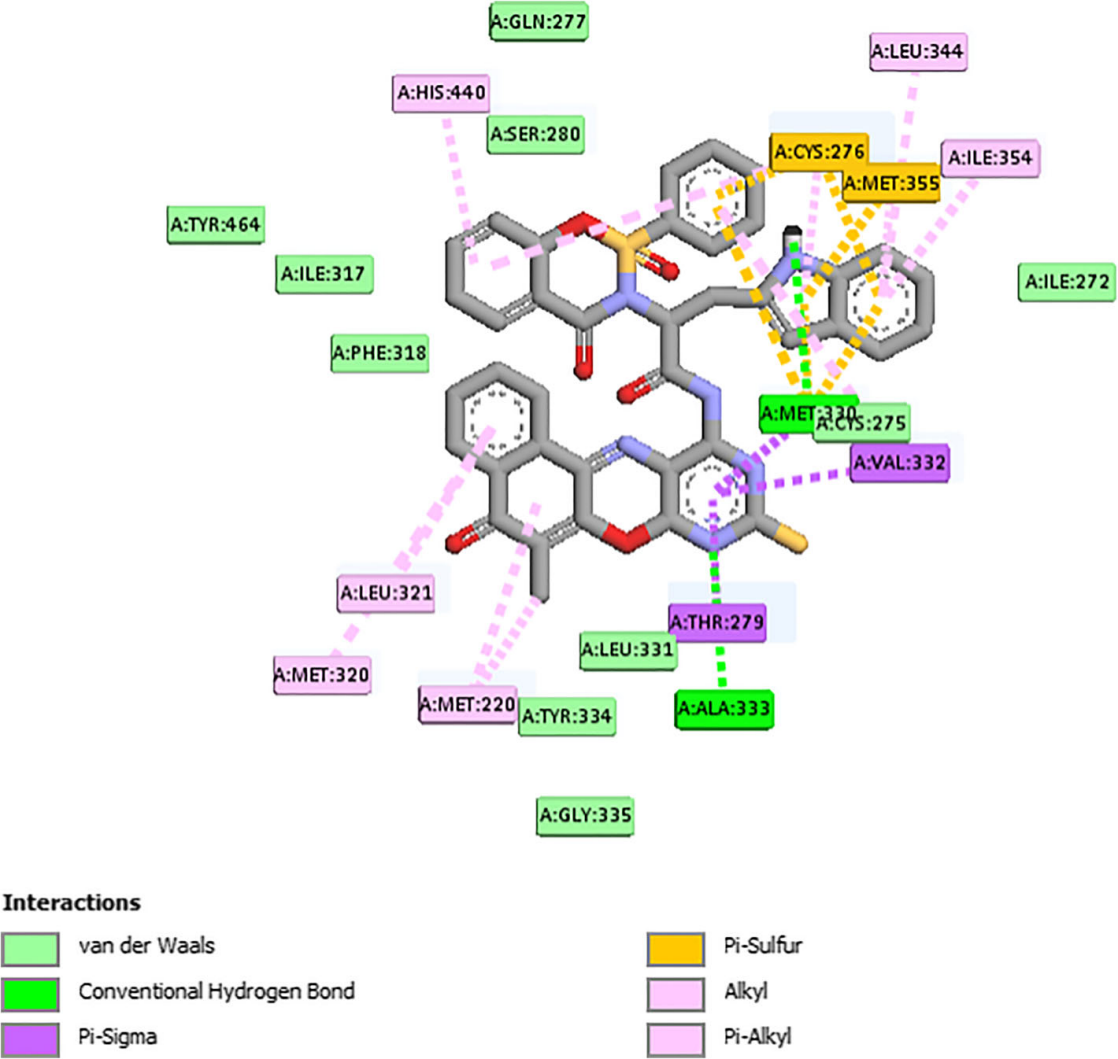
Types of interaction between the 9 k and 2P54

**Fig. 8:**
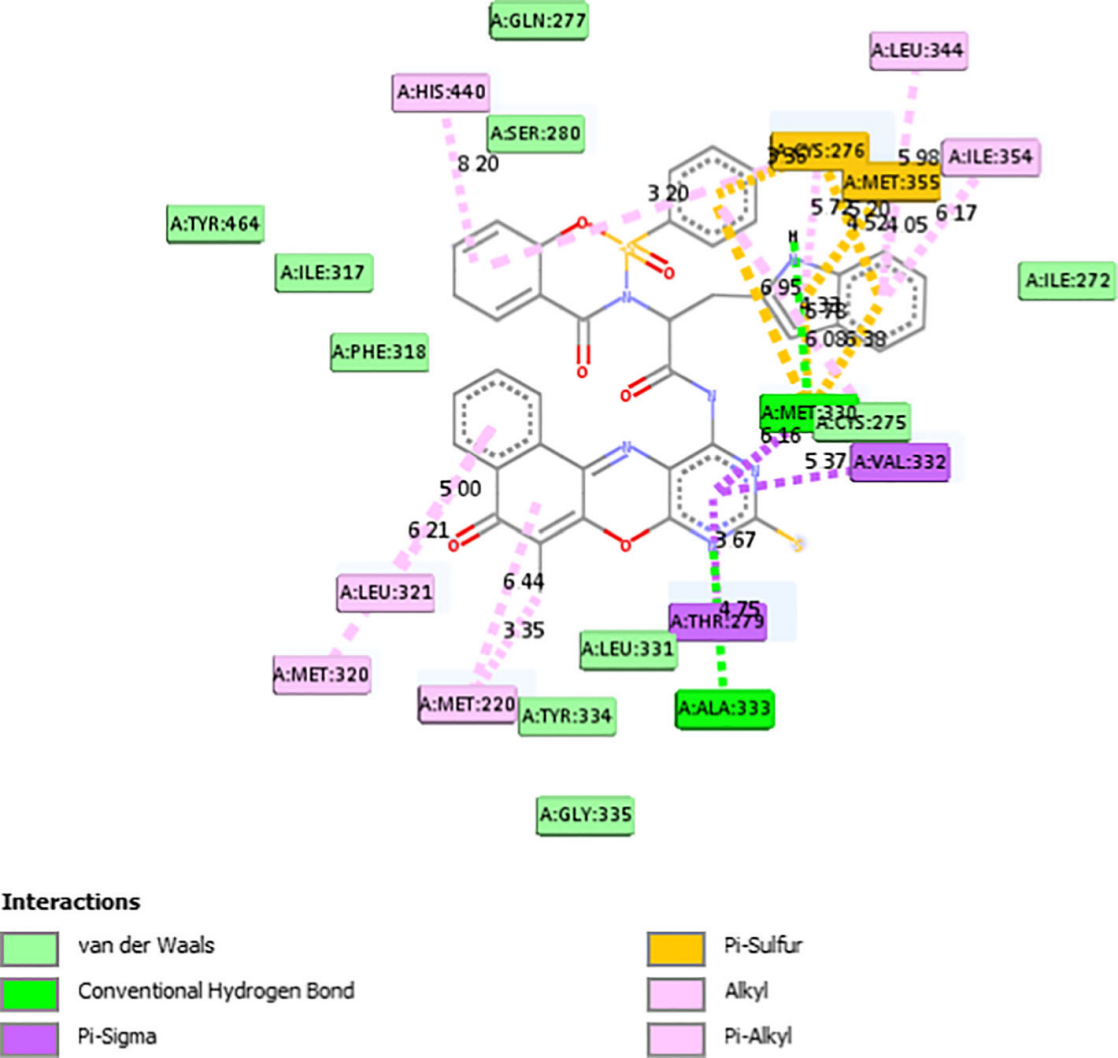
distance of interaction between 2P54 and 9 k in Å

**Fig. 9:**
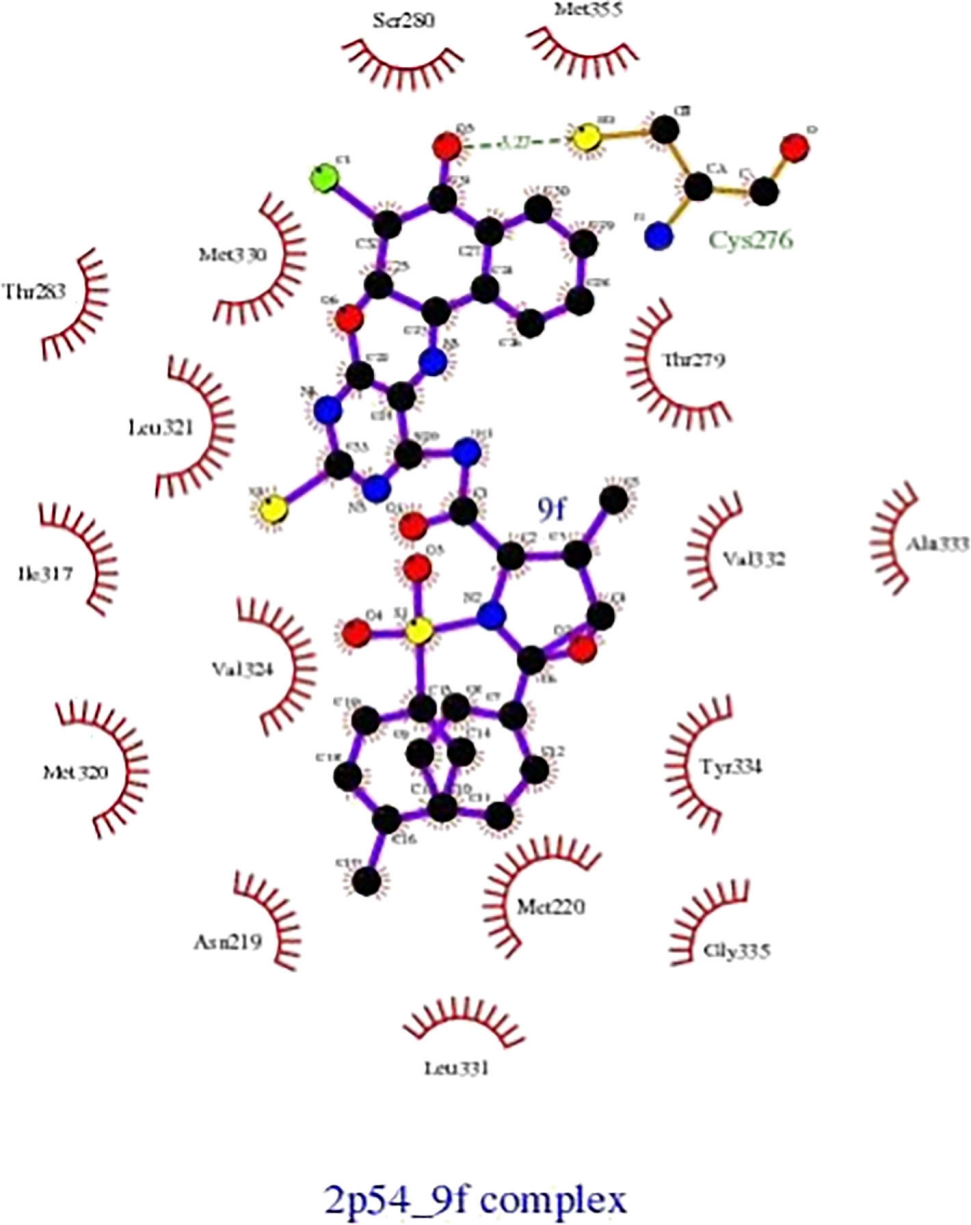
Ligplot^+^ results for 2p54_9f complex, showing all the 17 amino acid residues of active pocket

**Fig. 10:**
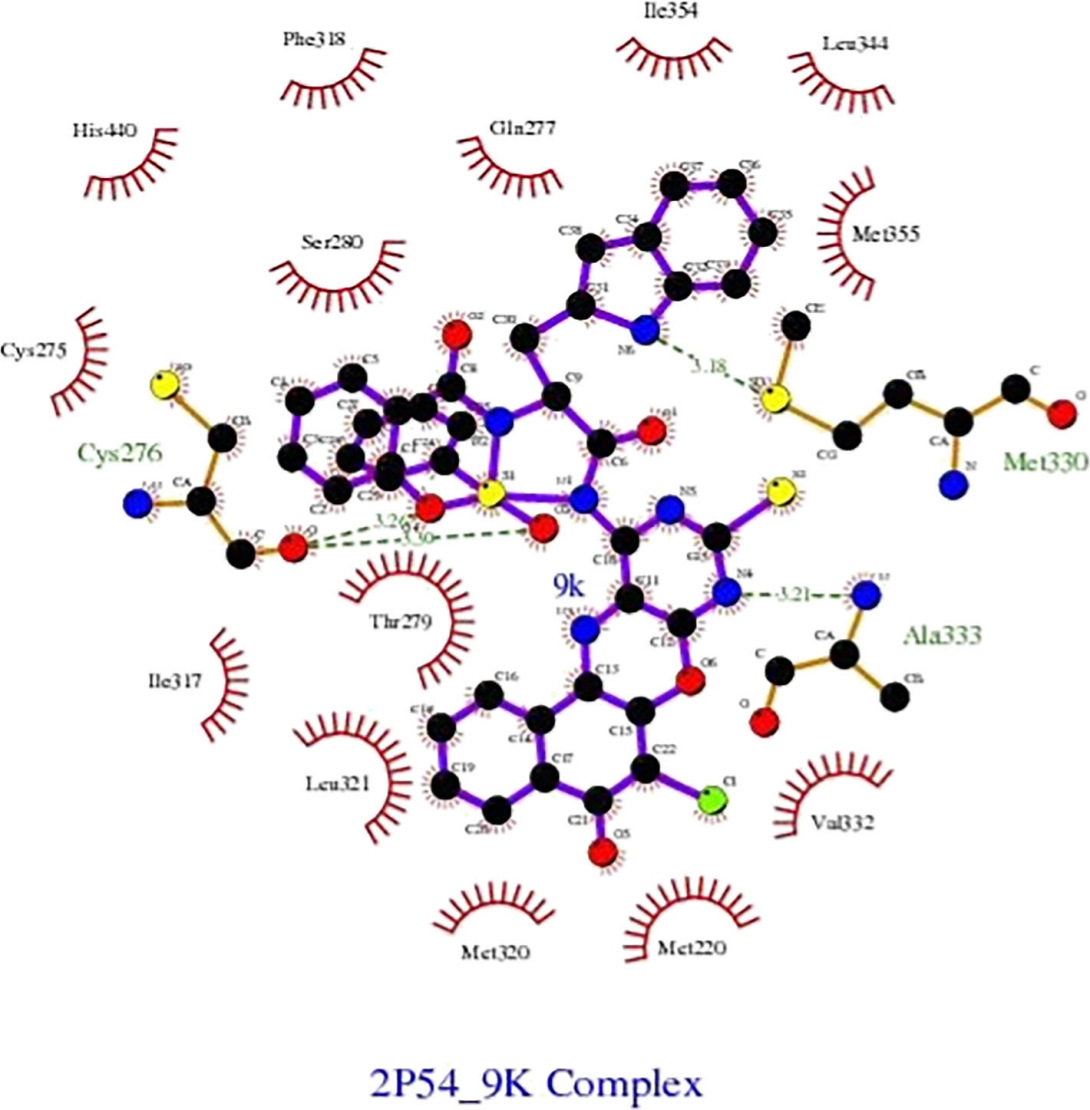
Ligplot^+^ results for 2p54_9 k complex, showing all the 17 amino acid residues of active pocket

**Fig. 11:**
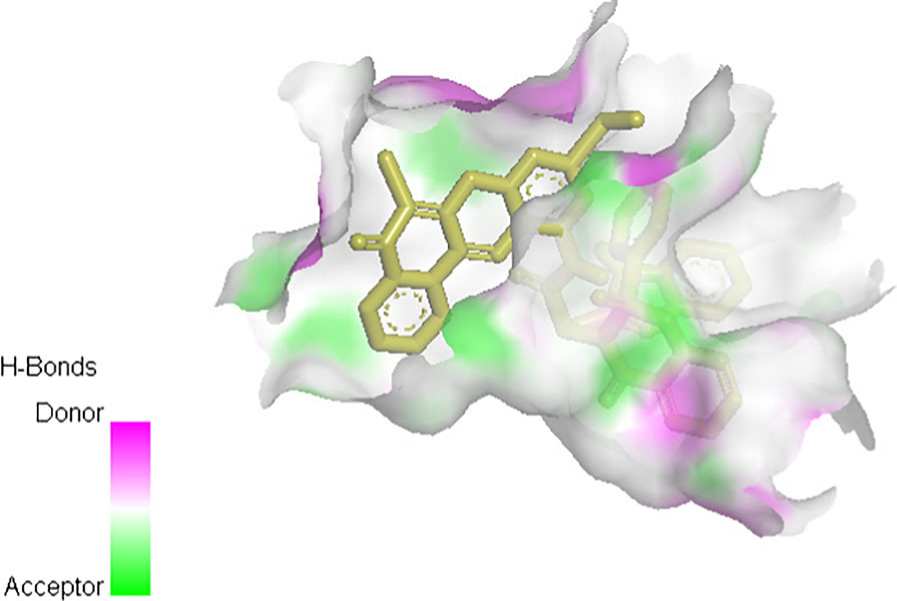
9 k ligand inside the protein pockets showing regions of H-Bond donors and acceptors

From Table 2, all the compounds showed significant binding affinities with the receptor used. The result revealed that compound **9f** showed higher binding affinities with the receptor than the other compounds assessed. The binding energy of **9f** with 2p54 is given as − 12.6 kcal/mol with an activity constant (*Ki*) of 2.8 nM. Therefore, we carried out further study on **9f** and **9 k** to show its binding modes in the receptor, **2p54** (Figs. 5 and 6) and to identify the different chemical interactions between **9f** and **9 k** with different amino acid residues of the receptor (Figs. 7 and 8). The ligand, **9f** and **9 k** were well fitted into the binding cavity of the receptor, 2p54 resulting in the observed binding energies. Because of these interactions, **9f** and **9 k** can elicit its pharmacological actions on the organisms as it competitively antagonizes the normal biochemical processes in the receptor. It can also be seen from Table 2, as also indicated in Figs.7 and 8 that four different chemical interactions were involved namely: hydrogen bonding, van der Waal, pi-sigma and pi-alkyl interactions. Figs. 7 and 8 and a **Ligplot**^**+**^ (Figs. 9 and 10) clearly suggest that a total of 17 active amino acid residues of 2p54 interacted with different atoms/groups of compound **9f** and **9 k** respectively. They are MET:355, SER:280, MET:330, THR:283, LEU:321, ILE:317, VAL:324, MET:320, THR:279, VAL:332, TYR:334, GLY:335, MET:220, LEU:331, ASP:219, ALA:333 and CYS:276 for 2p54_9f and ILE:354, LEU:344, MET:355, MET:330, ALA:333, VAL:332, MET:220, MET:320, LEU:321, THR:279, ILE:317, CYS:275, CYS:276, HIS:440, SER:280, PHE:318 and GLN:277 for 2p54_7k. Figure 11 shows the binding of compound **9 k** in the pocket of peroxisome proliferator activated receptor alpha (2p54).

### Lipid lowering activities

Cardiovascular disease (CVD) includes conditions such as heart failure, congenital heart disease, coronary heart disease, arrhythmia, stroke, heart valve disease and hypertension. Many epidemiological studies have shown that a direct correlation exists between lipid abnormalities particularly elevated levels of total cholesterol, triglycerides and low density lipoprotein cholesterol, and the risk of coronary vascular disease [2]. The in vivo lipid lowering activities (Table 1) showed that all the compounds prepared reduced the triglyceride (TG) and total cholesterol (C) content after 1 week. In some case, an increment in high density lipoprotein cholesterol (HDLC) were observed whereas there were observed decreased in the HDLC level in some cases as against the expectation of a good heart drug. The very low density lipoprotein cholesterol (VLDLC) values showed total decrease when compared with the control, although in some cases, the decrease was not significantly different. Some of the derivatives showed fascinating features of good heart drugs. Compounds **9a, 9f, 9j** and **9 k** showed marked decrease in the triglycerides contents from 176.2 mg/dL in the control to 148.2, 144.6, 149.9 and 146.4 mg/dL respectively. The total cholesterol decreased from 206.3 mg/dL in the control to 176.7, 173.1, 185.3 and 163.8 mg/dL respectively for compounds **9a, 9f, 9j** and **9 k.** Like the results of triglycerides and total cholesterol, there was marked decrease in the low density lipoprotein cholesterol and very low density lipoprotein cholesterol contents from 101.0 and 34.6 mg/dL in the control to 31.4 and 28.9 mg/dL for compound **9f** and 2.1 and 29.3 mg/dL for compound **9 k** respectively. Compounds **9a** and **9j** showed an increased LDLC value thereby marking them bad candidate for treatment of heart disease. As was the case for LDLC, only compounds **9f** and **9 k** showed marked increase in the HDLC level.

**Table 1:** Binding energy (ΔG - kcal/mol) of compounds with target proteins (2p54)

Comp	ΔG (kcal/mol) 2P54	Ligand efficiency
9a 9b 9c 9d 9e 9f 9 g 9 h 9i 9j 9 k 9 l 9 m 9n 9o	−8.4 − 7.9 − 7.8 − 7.5 − 8.4 − 12.6 − 7.7 − 8.2 − 9.6 − 8.4 − 10.8 − 8.9 − 8.3 − 7.5 − 7.8	0.19 0.15 0.14 0.16 0.18 0.27 0.19 0.21 0.22 0.17 0.20 0.19 0.18 0.19 0.21

The percentage lipid lowering activities of the new carboxamides were presented in Table 2. Compounds **9a, 9f** and **9 k** showed percentage reduction in triglyceride level of 17.3, 16.5 and 15.5% respectively which is comparable with gemfibrozil (16.1%, Table 3). Compounds **9d, 9e, 9f, 9i, 9o, 9 l, 9 m** and **9 k** reduced the total cholesterol level at 25.6, 17.0, 16.1, 17.7, 19.3, 22.5, 17.0, 20.6% respectively which were comparable with percentage reduction of 15.7% of gemfibrozil. Compounds **9d, 9f** and **9 k** increased the high density lipoprotein cholesterol level in the range of 41.7, 59.6 and 87.4% respectively comparable to 55.8% reduction from gemfibrozil. Compound **9 k** had 97.9% reduction of the low density lipoprotein cholesterol level against gemfibrozil (64.90%). Compound **9f** reduced very low density lipoprotein cholesterol level at 16.5% which was comparable with gemfibrozil (16.1%). The proline derivatives **9f, 9 h, 9n** and **9o** were the more active than the acyclic derivatives. The structure-activity relationship study showed that methyl group para to the benzenesulphonamide is significant in the lipid lowering activity. In exception of tryptophan derivatives (**9c** and **9 k**), the para substituted derivatives showed better lipid lowering activity than the derivatives without substitution (**9i-o**). This finding is also supported by the molecular docking experiment which showed an additional interaction between the methyl group and an amino acid in the protein (2p54) as shown in Fig. 9. Among the tryptophan derivatives **9 k** and **9c**, the additional presence of NH group is suggested to have affected the lipid lowering activity possibly by positioning compound **9 k** for better binding in a preferred orientation than the para substituted **9c** which is in agreement with the molecular docking results.

**Table 2:** in vivo lipid lowering activities

S/N	TG (mg/dL)	TC (mg/dL)	HDLC (mg/dL)	LDLC (mg/dL)	VLDLC (mg/dL)
9a	148.2	179.7	23.9	126.2	29.6
9b	156.3	176.2	45.2	99.8	31.3
9c	153.6	178.3	30.9	116.7	30.7
9d	153.6	153.5	100.1	22.6	30.7
9e	165.6	171.2	52.6	85.5	33.1
9f	144.6	173.1	112.8	31.4	28.9
9 g	157.7	166.6	85.6	49.4	31.6
9 h	157.3	179.7	55.2	93.0	31.5
9i	165.9	169.8	23.8	112.8	33.2
9j	149.9	185.3	19.2	136.1	29.9
9 k	146.4	163.8	132.4	2.1	29.3
9 l	159.3	159.9	45.3	82.7	31.9
9 m	157.3	171.2	73.9	65.9	31.5
9n	152.3	179.7	65.0	84.2	30.5
9o	157.7	166.6	85.6	49.4	31.6
gemfibrozil	145.2	174.6	110.1	35.5	29.1
control	173.2	206.3	70.7	101.0	34.6

**Table 3:** Percentages of lipid profile of the new derivatives

S/N	TG	TC	HDLC	LDLC	VLDLC
9a	14.4	12.9	–	−24.9	14.4
9b	9.7	14.6	–	1.3	9.8
9c	11.3	13.6	–	−15.5	11.3
9d	11.3	25.6	41.7	77.6	11.3
9e	4.4	17.0	–	15.3	4.4
9f	16.5	16.1	59.6	68.9	16.5
9 g	10.5	13.3	1.9	25.1	10.5
9 h	9.2	12.9	–	7.9	9.2
9i	4.2	17.7	–	−11.6	4.2
9j	13.4	10.2	–	−34.7	13.4
9 k	15.5	20.6	87.4	97.9	15.5
9 l	8.0	22.5	–	18.2	8.0
9 m	9.2	17.0	4.6	34.8	9.2
9n	9.2	12.9	–	16.7	12.1
9o	8.9	19.3	21.2	51.1	8.9
gemfibrozil	16.1	15.4	55.8	64.9	16.1

TG implies triglyceride; TC implies total cholesterol; HDLC imply high density lipoprotein cholesterol; LDLC implies low density lipoprotein cholesterol; VLDLC implies very low density lipoprotein cholesterol. The percentage triglyceride were calculated using TG (%) = [TG (control)-TG (sample)/TG (control)]X 100. This was also used for percentage total cholesterol, high density lipoprotein cholesterol, low density lipoprotein and very low density lipoprotein cholesterol

### Toxicity analysis

The result obtained from kidney function test (KFT) and liver function test (LFT) as presented in Table 4 showed that at 500 mg/kg administration, the new derivatives showed no significant change in the concentration of the biomarkers on comparison with the control. The LD_50_ values (Table 4) of the two most active carboxamide derivatives (**7f** and **7 k**) showed that they the new compounds had better toxicity profile when compared to gemfibrozil. In general, the KFT, LFT and LD_50_ values of the reported lipid lowering agents suggest that they are relatively safe toxicologically.

**Table 4:** LFT, KFT and LD_50_ results of the most active derivatives

Biomarker	AST (IU/L)	ALT (IU/L)	ALP (IU/L)	Urea (IU/L)	Creatinine (IU/L)	Uric acid (IU/L)	LD_50_ μM/kg
9f	41 ± 2.00	18.4 ± 0.80	0.8 ± 0.016	17.7 ± 0.008	1.6 ± 0.33	6.6 ± 0.02	8.0
9 k	42 ± 1.83	18.9 ± 0.42	0.7 ± 0.020	18.1 ± 0.002	1.4 ± 0.11	6.6 ± 0.03	7.6
control	40 ± 1.65	20.0 ± 0.75	0.8 ± 0.014	19.1 ± 0.008	1.5 ± 0.05	6.4 ± 0.005	8.8 [32]

All data were expressed as mean ± SE (standard error). AST means aspartate aminotransferase, ALT means alanine transaminase and ALP means alkaline phosphatase

## Conclusion

Fifteen new derivatives of phenoxazinone bearing sulphonamide and carboxamide pharmacophores have been described in this work. All the compounds showed good binding with PPAR alpha (2p54) with binding energies ranging from − 7.5 to 12.6 kcal/mol. Compound **9f** showed *Ki* of 2.8 nM and binding energy of 12.6 kcal/mol. All the derivatives showed lipid lowering activity in the in vivo experiment but only two derivatives **9f** and **9 k** showed characteristics of a lipid lowering agent comparable to statins and fibrates. The results of the in vivo experiment were in agreement with the in silico experiment as compounds **9f** and **9 k** were found to be the most active derivatives in both experiment. Although compound **9 k** was more active than **9f** in the in vivo test contrary to the result obtained from the in silico experiment, this observation is in agreement with the experimental error of docking procedure. The structure-activity relationship study showed that the 4-methylbenzenesulphonamide derivatives were better lipid lowering agent than the benzenesulphonamide derivatives in exception of compound **7 k** which was more active than its corresponding 4-methylbenzenesulphonamide derivative **7c.** The kidney function tests, liver function tests and LD_50_ showed that the compounds were safe as there was no remarkable change in the biomarkers of the mice fed with the compounds and the control.

## Abbreviations

ALP: Alkaline phosphatase
ALT: Alanine transaminase
AST: Aspartate aminotransferase
CHD: Coronary heart disease
CVD: Cardiovascular disease
ESI-TOF: Electrospray ionization-time of flight
FTIR: Fourier Transform Infrared
HDLC: High density lipoprotein cholesterol
HMG-CoA: 3-hydroxy-3-methylglutaryl-coenzyme A
HRMS: High resolution mass spectrometer
IIT: Indian Institute of Technology
KFT: Kidney function test
LD_50_: Lethal Dose at 50%
LDLC: Low density lipoprotein cholesterol
LFT: Liver Function test
LGA: Lamarckian genetic algorithm
NMR: Nuclear magnetic resonance
PDB: Protein data bank
PPAR-α: Peroxisome proliferator-activated receptor alpha
RCSB: Royal Chemical Society bank
TC: Total Cholesterol
TG: Triglycerides
VLDLC: Very low density lipoprotein cholesterol

## References

[1] Sarwar N, Danesh J, Eiriksdottir G, Sigurddson G, Wareham N, Bingham S, Boekholdt SM, Khaw KT, Gudnason V (2007). Circulation.

[2] Stone GW, Maehara A, Lansky AJ, de Bruyne B, Cristea E, Mintz GS, Mehran R, McPherson J, Farhat N, Marso SP, Parise H, Temphin B, White R, Zhang Z, Serruys PW (2011). N Engl J Med.

[3] Steinberg D (2006). J Lipid Res.

[4] Pahan K (2006). Cell Mol Life Sci.

[5] Jick H, Zornberg GL, Jick SS, Seshadri S, Drachman DA (2000). Lancet.

[6] Anderson KM, Odell PM, Wilson PW, Kannel WB (1991). Am Heart J.

[7] Malik S, Wong ND, Franklin SS, Kamath TV, L'Italien GJ, Pio JR (2004). Circulation.

[8] Malloy MJ, Kane JP (2001). Adv Intern Med.

[9] Thompson PD, Clarkson P, Karas RH (2003). JAMA.

[10] Furberg CD, Pitt B (2001). Curr Control Trials Cardiovasc Med.

[11] Ference BA, Majeed F, Penumetcha R, Flack JM, Brook RD (2015). J Am Coll Cardiol.

[12] Zynolebadi N, Moradi MN, Ghasemi H, Totonchi A, Goodarzi M, Oshaghi EA (2012). Res Pharm Sci.

[13] Elisaf M (2002). Curr Med Res Opin.

[14] Staels B, Dallongeville J, Auwerx J, Schoonjans K, Leitersdorf E, Fruchart JC (1998). Circulation.

[15] Willson TM, Brown PJ, Sternbach DD, Henke BR (2000). The PPARs: from orphan receptors to drug discovery. J Med Chem.

[16] Rubins HB, Robins SJ, Collins D, Fye CL, Anderson JW, Elam MB, Faas FH, Linares E, Schaefer EJ, Schectman G (1999). N Engl J Med.

[17] Lazarow PB, Shio H, Leroy-Houyet MA, Lipid J (1982). Res.

[18] Gray TJ, Beamand JA, Lake BG, Foster JR, Gangolli SD (1982). Toxicol Letter.

[19] Reddy JK, Krishnakantha TP (1975). Science.

[20] Leighton F, Coloma L, Koenig C (1975). J Cell Biol.

[21] Rao MS, Subbarao V, Reddy JK, Natl Cancer J (1986). Inst.

[22] Ugwu DI, Okoro UC, Ukoha PO, Okafor S, Ibezim A, Kumar NM (2017). Eur J Med Chem.

[23] Morris GM, Goodsell DS, Halliday RS, Huey R, Hart WE, Belew RK, Olson AJ (1998). J Comput Chem.

[24] Mohammadi A, Norouzian P, Jamshidi M, Najafi N, Oshaghi EA (2013). J Adv Chem.

[25] Allain CC, Poon LS, Chan CSG, Richmond W, Fu PC (1974). Clin Chem.

[26] Albers JJ, Warnick GR, Chenng MC (1978). Lipids.

[27] Demacker PN, Hijmans AG, Brennink BJ, Jansen BP, Vantlaar A (1984). Clin Chem.

[28] Reitman S, Frankel S (1957). Am J Clin Pathol.

[29] Kaplan A, Teng LL. In selected methods of clinical chemistry, Ed. By W.R. Faulkner and S. Meites, Washington: AACC. 1982;9:357–63.

[30] Ochei J, Kolhatkar A. Medical Laboratory Science. Theory and Practice. 2nd Edition. New Delhi: Tata Mcgraw-Hill; 2000. p. 331–49.

[31] Ghosh M. Fundamentals of experimental pharmacology scientific book agency. Calcutta. 1984:153–8.

[32] Pfizer Material Safety Data Sheet (2006) 4 www.pfizer.com/files/products/material_ safety_data/500.pdf assessed on 09/08/2017. Accessed 9 Aug 2017.

